# Malaria vaccination in Africa: A mini-review of challenges and opportunities

**DOI:** 10.1097/MD.0000000000038565

**Published:** 2024-06-14

**Authors:** David B. Olawade, Ojima Z. Wada, Chiamaka Norah Ezeagu, Nicholas Aderinto, Malik A. Balogun, Fiyinfoluwa T. Asaolu, Aanuoluwapo Clement David-Olawade

**Affiliations:** a Department of Allied and Public Health, School of Health, Sport and Bioscience, University of East London, London, UK; b Division of Sustainable Development, College of Science and Engineering, Hamad Bin Khalifa University, Qatar Foundation, Doha, Qatar; c Department of Public Health, School of Health and Life Sciences, Teesside University, Middlesbrough, UK; d Department of Medicine and Surgery, Ladoke Akintola University of Technology, Ogbomoso, Nigeria; e Department of Public Health, University of Suffolk, UK; f Department of Biomedical Science, De Montfort University, UK; g Department of Nursing, University of Derby, Derby, UK.

**Keywords:** Africa, malaria vaccine, vaccination, vaccine hesitancy

## Abstract

Malaria remains an endemic public health concern in Africa, significantly contributing to morbidity and mortality rates. The inadequacies of traditional prevention measures, like integrated vector management and antimalarial drugs, have spurred efforts to strengthen the development and deployment of malaria vaccines. In addition to existing interventions like insecticide-treated bed nets and artemisinin-based combination therapies, malaria vaccine introduction and implementation in Africa could drastically reduce the disease burden and hasten steps toward malaria elimination. The malaria vaccine rollout is imminent as optimistic results from final clinical trials are anticipated. Thus, determining potential hurdles to malaria vaccine delivery and uptake in malaria-endemic regions of sub-Saharan Africa will enhance decisions and policymakers’ preparedness to facilitate efficient and equitable vaccine delivery. A multisectoral approach is recommended to increase funding and resources, active community engagement and participation, and the involvement of healthcare providers.

## 1. Introduction

Malaria remains one of the leading causes of under-5 morbidity and mortality in sub-Saharan Africa (SSA). All efforts targeted at malaria elimination aim to achieve Sustainable Development Goal 3.3.3, which envisions the reduction of malaria incidence and mortality by 90% from 2015 to 2030.^[1]^ Despite immense efforts by government health agencies and international organizations like the World Health Organization (WHO) to curb the disease, globally malaria-related morbidity and mortality increased by 6% and 12%, respectively, between 2019 and 2020.^[2]^ The global effort to curb the transmission of malaria and prevent associated deaths resulted in the initiation of the “Malaria Vaccine Technology Roadmap,” which aims to license vaccines that can protect against clinical malaria caused by Plasmodium falciparum and Plasmodium vivax with the efficacy of at least 75%, as well as to develop vaccines aimed at reducing the transmission of the malaria parasite, eventually lowering the incidence rate.^[3]^

In October 2021, the WHO recommended the widespread delivery of the malaria vaccine RTS,S/AS01, among at-risk children in SSA and selected areas with significant *P falciparum* malaria transmission.^[4]^ The vaccine had proved effective against malaria transmitted by *P falciparum* in Phase 1 and 2 clinical trials.^[5]^ Four vaccine doses were recommended for children from their fifth month.^[6]^ By the end of 2021, around 2.5 million doses of the malaria vaccine had been administered in Malawi, Ghana, and Kenya, recording a high safety profile and significantly reducing severe malaria by 30%.^[7]^ However, during Phase 3 clinical trials, the vaccine’s overall efficacy was much lower- around 36% among children between 5 and 17 months and 26% among babies between 6 and 12 weeks.^[5,8]^ In fact, in one study, the protective efficacy of combined chemoprevention and RTS,S resulted in substantially fewer clinical malaria cases per year (113 per 1000 children) compared to sole vaccination usage (278 per 1000 children) and sole chemoprevention with sulfadoxine-pyrimethamine and amodiaquine (305 per 1000 children).^[9]^ Such efficacy rates show a need for vaccine improvement to reach the 75% vaccine efficacy threshold set in the Malaria Vaccine Technology Roadmap.

In September 2022, researchers reported the development of a malaria vaccine- R21, which improved the RTS,S vaccine.^[10]^ Early clinical trials, including 409 children in Burkina Faso, revealed the vaccine had an efficacy as high as 80% among the high-dose adjuvant group and 71% among the low-dose adjuvant group after delivering the primary 3-dose regimen and a booster dose.^[10]^ With the optimistic likelihood of the vaccine attaining a high efficacy (≥75%), it is hoped that there will be a massive malaria vaccine rollout across SSA in the coming years. Thus, examining the potential impediments to fast and efficient vaccine delivery in SSA is integral.^[11,12]^ Therefore, this mini-review studies the potential barriers that might forestall the efforts to facilitate the fast, efficient, and equitable delivery of malaria vaccines in SSA.

## 2. Methods

A comprehensive literature search was conducted to identify pertinent articles focusing on the impediments and challenges associated with the delivery and acceptance of malaria vaccines in SSA. Utilizing electronic databases such as PubMed/MEDLINE, Scopus, Web of Science, and Google Scholar, the search encompassed articles published in English from January 2010 to December 2023. Keywords including “malaria vaccine,” “Africa,” “vaccination,” “vaccine hesitancy,” and “malaria-endemic regions” were employed to refine the search results. The inclusion criteria encompassed studies that elucidated barriers and hurdles to malaria vaccine delivery and uptake specifically within sub-Saharan African contexts, including original research, systematic reviews, meta-analyses, and qualitative studies. Articles were excluded if they did not directly address the topic, were published in languages other than English, were duplicates, or lacked full-text accessibility.

The extracted data were subjected to thematic analysis, facilitating the identification of common barriers and challenges associated with malaria vaccine delivery and uptake in SSA. Through thematic grouping of extracted data, common themes and subthemes emerged, allowing for a comprehensive understanding of the multifaceted challenges hindering effective malaria vaccine implementation in the region. The findings were synthesized narratively, providing valuable insights to inform subsequent discussions and recommendations within the review. Overall, the approach adopted in this mini-review ensured a robust and comprehensive exploration of the barriers and challenges impeding the delivery and uptake of malaria vaccines in SSA.

### 2.1. Malaria vaccination coverage in Africa

Despite accounting for the highest burden of disease, and concerted partnership and collaboration efforts by governments, NGOs, and the private sector to advance malaria vaccine development and rollout, only 5% of people in Africa have received malaria vaccination.^[13]^ This was largely influenced by the coronavirus disease 2019 (COVID-19) pandemic, which disrupted malaria vaccination rollout in Africa. Notably, the sudden insurgence of COVID-19 caused a major shift in medical resources and financial investments from malaria control to tackling the pandemic.^[14]^ However, the WHO Global Technical Strategy for Malaria (2016–2030) aims to accelerate the widespread deployment of malaria vaccines in endemic countries to achieve at least a 75% reduction in global malaria cases and deaths by 2025.^[15]^

Although information on malaria vaccination coverage is lacking for most African countries, likely, the vaccination rates vary widely across countries and geographical areas.^[16]^ Ghana, Kenya, Malawi, and Mozambique are the few African nations that have commenced malaria vaccine pilot projects and small-scale implementation studies. These studies have a cumulative coverage rate of >1 million children.^[17,18]^ While this is impressive, this figure is quite small relative to the disease burden. Of these countries, Malawi and Ghana have incorporated the RTS,S vaccine into their immunization programs.^[19]^ For example, the pilot program for the RTS,S/AS01 vaccine in Ghana, has shown promising vaccine uptake and a significant reduction in malaria cases among vaccinated children.^[20,21]^ Ghana was also the first African country, followed by Nigeria, to approve the malaria vaccine R21/matrix-M, having demonstrated a high-level efficacy of 77%.^[22,23]^

### 2.2. Overview of the challenges in Africa

Several studies have attempted to highlight potential challenges in upscaling the malaria vaccination uptake and coverage in Africa. Studies in Ghana, Burkina Faso, and Nigeria respectively reported problems including inadequate/lack of community engagement and education to promote vaccine uptake and adherence,^[20]^ insufficient funding and political commitment to ensure the successful implementation and scale-up of malaria vaccination programs,^[24]^ poor vaccine availability, inadequate funding and lack of awareness.^[25]^ These studies indicate a range of cross-cutting challenges to successful malaria vaccination programs.

#### 2.2.1. Limited access

Although malaria vaccines have shown promising results in clinical trials, their implementation and scaling-up have faced significant challenges. A major obstacle to improving malaria vaccine coverage in Africa is the lack of vaccine accessibility.^[22]^ Many African nations lack the infrastructure, like good remote access roads, and resources to effectively deliver and distribute vaccines across rural areas where malaria is endemic. Other issues include limited transportation options, logistics issues, and poor road networks. Unfavorable weather and geographical conditions hinder immunization activities, particularly in the tropics and mountainous areas.^[26]^

Likewise, the lack of a solid cold chain system, necessary to maintain the potency and safety of vaccinations by keeping them at the correct temperature, may impede vaccine accessibility. Some African nations lack the facilities, tools, and trained staff required to maintain a cold chain system, increasing stock expiration.^[27]^ Moreover, conflict-related insecurities could further make it difficult to deliver vaccines to some areas.^[28]^ Lack or inadequate access to malaria vaccines is therefore likely to undermine efforts to reduce the malaria burden in Africa.

#### 2.2.2. Limited data

The lack of data makes it challenging to evaluate malaria vaccination coverage in Africa accurately^[29]^ or evaluate how vaccination affects morbidity and mortality.^[30]^ Finding low-coverage locations and comprehending the causes of these gaps may be difficult without precise and current data. One reason for the limited amount of data available is the lack of a reliable surveillance method to track malaria vaccination coverage.^[31]^ Many African countries have limited infrastructure and resources necessary to conduct frequent surveys and gather data on vaccine coverage.^[32,33]^ Poor or missing data makes developing policies and tracking their effectiveness very challenging.

The lack of comprehensive data on malaria vaccine efficacy also complicates decision-making on funding and implementing vaccination campaigns.^[34,35]^ More thorough data are needed on the effectiveness of malaria vaccines in real-world situations rather than just pilot or demonstration projects.^[5]^ Further data are also needed on the cost-effectiveness of malaria vaccination programs in Africa, without which convincing donors and policymakers for funding and other resource allocation is much more difficult.^[36]^

#### 2.2.3. Lack of awareness

Lack of knowledge about malaria vaccinations is likely to have harmed vaccination rates in Africa.^[37]^ Many Africans are unaware of the benefits of vaccination against malaria or the fact that malaria vaccinations are available.^[38,39]^ Many African nations lack the resources necessary to educate and inform the public about malaria vaccinations,^[35]^ especially in rural and distant areas where access to healthcare is poor. Furthermore, access to trustworthy health information is often limited and if people find it challenging to comprehend the value of malaria vaccines then they are unlikely to explore how to obtain them.^[40]^

Lack of knowledge is linked to deficient community sensitization and interactions. Many African malaria vaccination campaigns are developed without the involvement and cooperation of the local communities.^[41]^ Hence, vaccines may not be well-received and have low levels of trust. Most of the participants in a study conducted in Ghana had little to no understanding of malaria vaccines and the disease’s prevention.^[38,42]^ Minimal media attention and public education initiatives on malaria vaccinations also contribute to limited population awareness of malaria vaccines.^[43]^ Partly this is due to a lack of funding for large public awareness campaigns about malaria vaccines. A lack of political support and campaign funding may also be a factor.^[44]^

#### 2.2.4. Limited funding

Adequate funding is critical for vaccine development and distribution.^[45,46]^ It is also needed to improve access, awareness, health workforce, and data collection. Recent efforts to address the challenges of malaria vaccination in Africa have focused on improving funding and resources for vaccination campaigns.^[47]^ Malaria vaccination campaigns can be costly, and many African nations lack the means to properly finance them.^[48]^ Restricted access to healthcare, particularly in rural and distant locations, contributes to limited vaccines.

Significant financial investment is needed to successfully implement malaria vaccination programs in Africa to attain high coverage.^[49]^ Success requires resources, funding, education and training, data collection and analysis, and close community collaboration. The WHO has developed a global vaccine action plan to increase access to vaccines and immunization programs, particularly in low-income countries.^[50]^ The plan includes initiatives such as the Gavi Vaccine Alliance, which provides funding and resources for vaccination programs in developing countries.^[51]^ African countries can leverage such funding to improve malaria vaccination implementation programs.

#### 2.2.5. Limited health insurance

In the last decade, substantial workforce shortages have been reported across SSA.^[52]^ Improving malaria vaccination coverage in Africa would be problematic even with an available, adequate, and competent health workforce. It may be challenging to administer vaccines to inform and educate the public due to the lack of skilled healthcare workers, particularly in rural and isolated locations.^[53]^ Additionally, this may make it challenging to track and assess the accessibility and efficiency of programs to prevent malaria.^[54]^ The shortage of skilled medical personnel to give vaccinations results in longer waiting times and may consequently discourage vaccination-seeking behavior. Shortages also decrease the opportunity for awareness outreach and education efforts.^[55]^

The lack of a workforce to preserve vaccine potency and monitor and follow up vaccinated individuals, especially in remote areas may compromise vaccination coverage.^[56]^ Similarly, this personnel shortage also increases the workload of the few available ones, resulting in stress and high staff turnover. The development and application of successful solutions to increase coverage are also challenged by health workforce shortages. Healthcare providers, including community health workers, must also be trained and supported to effectively deliver malaria vaccines and address concerns and misconceptions about vaccinations.

#### 2.2.6. Complexity of malaria transmission

Vector Bionomics is one of the causes of complexity in malaria transmission. Changes in species composition, biting behavior, and environmental modifications can influence transmission and consequently result in differing levels of transmission in various locations.^[57]^ Other factors include seasonality, climate, and the presence of dissimilar malaria vectors.^[58]^ Effective malaria vaccination programs adapted to particular transmission patterns and environments may be challenging to develop and implement. For instance, as malaria is more prevalent in some places during certain months of the year than in others, it can be difficult to choose the best time for vaccination campaigns and to ensure that people receive the vaccine at the proper time to offer the greatest protection. While it is true that countries have varying malaria seasons, vaccination should take place in the off-peak seasons. Considering a pattern in high peak seasons for most African countries: Malawi (November–April), Burkina Faso (October–April), and Nigeria (August–October), ideally, vaccinations should be after April, but before August.

The existence of several malaria vectors and the genetic variety of the Plasmodium parasites that cause malaria also impact malaria transmission. This implies that developing vaccines effective against the many parasite and vector strains found in Africa may be challenging. Furthermore, separating the influence of the vaccine from other factors that influence malaria transmission such as the use of bed nets or other malaria control interventions, makes it difficult to assess the effectiveness of malaria vaccines.

#### 2.2.7. Vaccine hesitancy

Vaccine hesitancy describes an unwillingness to accept available vaccines. Learning from the COVID-19 era, several millions worldwide, particularly in Africa, were hesitant to receive the COVID-19 vaccine. Vaccine hesitancy is not limited to COVID-19 vaccines,^[58,59]^ and likely, decisions related to malaria vaccination will also be affected. Lack of confidence in the vaccination and the healthcare system, misinformation, rumors, and religious or cultural views^[60]^ are only a few causes of vaccine hesitation.^[61]^ Misinformation and misconceptions about the safety and efficacy of vaccines are frequently propagated via social media, religious or cultural leaders, and other sources.^[62]^ Although this is yet to be examined, malaria vaccines may also face considerable hesitancy across African nations. Policymakers and other important stakeholders may need to draw lessons from COVID-19 to tackle hesitancy and advance malaria vaccine coverage in Africa.

### 2.3. Multisectoral approach to malaria vaccination in Africa

Addressing the challenges of malaria vaccination in Africa requires a multisectoral approach that involves close collaboration between governments, nongovernmental organizations, healthcare providers, and communities.^[63]^ Governments must prioritize malaria vaccination programs and allocate adequate resources and funding to ensure successful implementation. Nongovernmental organizations are critical in supporting vaccination programs in Africa, particularly in areas with limited government resources. They can provide funding, technical assistance, and advocacy support to increase vaccine uptake and coverage. Community participation and engagement and increased health education on the malaria vaccine would promote positive public perceptions and improve vaccine acceptance within communities.

Furthermore, establishing the African Vaccine Manufacturing Initiative in 2010 aims to increase the availability of vaccines in Africa by developing local vaccine manufacturing capacity. This initiative recognizes the importance of local manufacturing in ensuring sustainable access to vaccines and reducing dependence on external sources. Another initiative, the Malaria Vaccine Implementation Program, is a collaboration between the WHO, the PATH Malaria Vaccine Initiative, and GSK, the manufacturer of the RTS,S/AS01 vaccine.^[64]^ The Malaria Vaccine Implementation Program aims to accelerate the deployment of the malaria vaccine in SSA and evaluate its impact on reducing malaria cases and deaths.

## 3. Conclusion

Inadequate malaria vaccine awareness in Africa is a complex issue influenced by many variables, including limited access to education and information, limited media coverage, a lack of political commitment, vaccine availability, and community involvement. A comprehensive and multisectoral effort is, thus, needed to address these issues. Increased funding for malaria vaccination programs, comprehensive public awareness campaigns about malaria vaccines, and community involvement in program development, implementation, and monitoring are necessary to solve these issues. To increase political commitment and funding in malaria immunization programs, it is also crucial to collaborate closely with national and regional governments.

Consequently, addressing the challenges of malaria vaccination in Africa requires a comprehensive and multisectoral approach that must aggregate efforts from national governments, international actors, private sector, and institutional think tanks to increase funding and resources, promote community awareness, engagement, and participation, leverage new vaccine technologies and address logistic complexities. With sustained efforts and collaboration, it is possible to overcome the malaria vaccine uptake and coverage challenges in Africa and, ultimately, reduce the burden of malaria on the continent.

## 4. Recommendations

The introduction and widespread adoption of malaria vaccines in Africa necessitates a concerted effort from various sectors to address existing challenges. Based on the identified barriers and complexities, the following recommendations are proposed to enhance the delivery and uptake of malaria vaccines in malaria-endemic regions of SSA:

1. Increased funding and resources: Governments, international organizations, and philanthropic entities should allocate more financial resources to malaria vaccination programs. Adequate funding is crucial for vaccine development, distribution, education, training of healthcare workers, and infrastructure development, including the establishment of robust cold chain systems. Collaboration with global initiatives like the Gavi Vaccine Alliance can help mobilize additional resources for malaria vaccination campaigns.
2. Active community engagement and participation: Community involvement is vital for the success of malaria vaccination programs. Efforts should be made to engage communities at every stage, from program planning and implementation to monitoring and evaluation. Community leaders, healthcare workers, and local volunteers can play key roles in promoting vaccine awareness, addressing misconceptions, and fostering trust in vaccines. Tailored communication strategies, including community meetings, door-to-door campaigns, and multimedia outreach, should be employed to ensure widespread understanding and acceptance of malaria vaccines.
3. Involvement of healthcare providers: Healthcare providers serve as trusted sources of health information and play a critical role in influencing vaccination decisions. Training programs should be conducted to equip healthcare workers with the knowledge and skills necessary to administer malaria vaccines effectively. Providers should also be trained in vaccine communication techniques to address vaccine hesitancy and concerns among patients and caregivers. Strengthening the healthcare workforce, particularly in rural and underserved areas, is essential for expanding vaccine coverage and access.
4. Multisectoral collaboration: Addressing the multifaceted challenges of malaria vaccination requires collaboration across sectors, including government agencies, nongovernmental organizations, academia, industry, and community-based organizations. A multisectoral approach can leverage diverse expertise, resources, and networks to develop innovative solutions and overcome logistical barriers. Platforms for knowledge sharing, coordination, and advocacy should be established to facilitate collaboration among stakeholders and ensure alignment with national and global health priorities.
5. Utilization of new vaccine technologies: Advances in vaccine technologies, such as the development of novel adjuvants and delivery systems, hold promise for improving the efficacy, safety, and accessibility of malaria vaccines. Continued investment in research and development is needed to accelerate the development of next-generation malaria vaccines with enhanced efficacy and durability. Collaboration between academia, industry, and public health agencies can expedite the translation of scientific discoveries into actionable interventions.
6. Addressing logistic complexities: The complex logistics of vaccine delivery in Africa, including transportation, storage, and distribution, pose significant challenges to vaccine access and coverage. Innovative approaches, such as mobile vaccine clinics, drone delivery systems, and decentralized vaccine distribution networks, should be explored to overcome logistical barriers and reach remote and underserved communities. Investment in infrastructure development and strengthening supply chain management systems is essential for ensuring the reliable and timely delivery of vaccines to the last mile.

## Author contributions

**Conceptualization:** David B. Olawade.

**Writing – original draft:** David B. Olawade, Ojima Z. Wada, Norah C. Ezeagu, Nicholas Aderinto, Malik A. Balogun, Fiyinfoluwa T. Asaolu, Aanuoluwapo Clement David-Olawade.

**Writing – review & editing:** David B. Olawade, Ojima Z. Wada, Norah C. Ezeagu, Nicholas Aderinto, Malik A. Balogun, Fiyinfoluwa T. Asaolu, Aanuoluwapo Clement David-Olawade.
